# Development and Characterization of Craft Beers Prepared with the Use of Tamarind (*Tamarindus indica* L.) Pulp

**DOI:** 10.3390/foods15010102

**Published:** 2025-12-29

**Authors:** Augusto de Souza da Silva, Hannah Caroline Santos Araujo, Mônica Silva de Jesus, Mario Jirlanio Guilherme Santos, Fernando José Castilho, Rafael Donizete Dutra Sandes, Daniel Alfonso Spudeit, Marcos dos Santos Lima, Maria Terezinha Santos Leite Neta, Narendra Narain

**Affiliations:** 1 PROCTA—Post Graduate Program in Food Science and Technology, Federal University of Sergipe, Av. Marcelo Déda Chagas, s/n, Jardim Rosa Elze, São Cristóvão 49100-000, SE, Brazil; 2 Laboratory of Flavor and Chromatographic Analysis, Federal University of Sergipe, Av. Marcelo Déda Chagas, s/n, Jardim Rosa Elze, São Cristóvão 49100-000, SE, Brazil; 3 Post Graduate Program in Biotechnology, Federal University of Sergipe, Northeast Biotechnology Network (RENORBIO), Av. Marcelo Déda Chagas, s/n, Jardim Rosa Elze, São Cristóvão 49107-230, SE, Brazil; 4 Department of Food Technology, Federal Institute of Educational Science and Technology Sertão Pernambucano, Campus Petrolina, Rod. BR 407 Km 08, S/N, Jardim São Paulo, Petrolina 56314-520, PE, Brazil; 5 Department of Food Technology, Federal University of Sergipe, Av. Marcelo Déda Chagas, s/n, Jardim Rosa Elze, São Cristóvão 49107-230, SE, Brazil

**Keywords:** beer, tamarind, volatile compounds, sensory analysis

## Abstract

This study evaluated the effects of tamarind pulp addition at different processing stages and concentrations on the physicochemical, volatile, bioactive, and sensory characteristics of Saison-style beers. The experiment was conducted in two stages. First, tamarind pulp (15 g/L) was added during fermentation or maturation, with maturation resulting in superior sensory scores and higher purchase intention (75%). In the second stage, tamarind pulp was added during maturation at 20, 40, and 60 g/L. The beers presented alcohol contents between 7.1 and 7.6% (*v*/*v*), bitterness values of 29–31 IBU, and color typical of the Saison style. Sensory analysis showed that the beer with 20 g/L of tamarind pulp achieved the best balance of acidity, aroma, and flavor, with acceptability indices above 70%, while higher concentrations increased residual acidity and reduced flavor acceptance. Chromatographic analyses indicated increased levels of esters and terpenes, particularly ethyl octanoate, phenethyl acetate, citronellol, and linalool, contributing fruity, floral, and citrus notes. Overall, tamarind pulp addition during maturation, especially at 20 g/L, improved the sensory and chemical profile of Saison beers, supporting its use as a craft beer adjunct.

## 1. Introduction

Beer is the world’s third most consumed beverage, after water and tea, and has a large global market [1]. Brazil currently ranks third worldwide in beer production, behind only the United States and China [2]. This expansion is believed to have had an impact on the development of Brazil’s craft beer industry. According to MAPA (Ministry of Agriculture and Livestock), the number of breweries registered in Brazil grew by 6.8% in 2023, with a total of 1847 breweries [3]. The increase in the consumption of craft beers may also be related to consumers’ search for new products that have a strong sensory appeal, differentiated flavors, and high quality at a reasonable price [4].

Craft beer is characterized by being a drink produced in small breweries, using different methods or adding different ingredients, such as the addition of fruits, herbs or alternative fermentable substrates, resulting in unique products [1]. Among craft beers, the Saison Ale type stands out for being a beer that has a low body, medium bitterness, and an acidic, fruity and spicy aroma and flavor. These sensory characteristics of this type of beer make it favorable for adding tropical fruits in its formulation. The growing demand for these beverages has renewed consumer interest in fruit beers.

These beverages can be classified into three types: those with fruits added directly, those with added fruit extract and those with added fruit flavor additives [5]. Although the addition of fruits increases the production costs of these beverages, it has the benefit of pleasing the consumer in terms of sensory requirements [6]. Incorporating fruit into the different stages like fermentation and maturation in beer production results in changes in its attributes, such as color, aroma and flavor. The addition of fruit at the boiling stage affects the flavor of the fruit due to the high temperature, while the addition of fruit during fermentation or maturation directly affects the formation of the specific fruit flavor [7].

Several types of fruits can be added to beer, such as peaches, mangoes, apples, pears, strawberries, blueberries, and especially tropical fruits, such as pineapple, banana, passion fruit, soursop, etc. [1,5]. Since tropical fruits have a characteristic acidic flavor, they tend to harmonize with the characteristics of craft beers, including Saison Ale type beers [8]. Several studies explore the incorporation of fruits into different types of beer [1,9,10,11]. Among the tropical fruits that can be added to beer is tamarind, a commercially valuable fruit cultivated in Asia, Africa, and the Americas. The tamarind fruit is typically composed of 55% pulp, 34% seed and 11% shell. Among the parts of the tamarind, the pulp stands out due to its contents of bioactive compounds, possessing antioxidants and antimicrobial properties. In addition to these properties, the pulp contains several organic acids, such as tartaric, acetic, formic and malic [12,13]. In the literature, some publications are found incorporating tamarind pulp as a flavoring agent [14,15,16]. However, despite these attributes, the application of tamarind pulp as a flavoring and nutritional ingredient in fermented alcoholic beverages, particularly beer, remains scarcely explored, highlighting a clear gap in the current literature.

In view of the above, this work explores the addition of tamarind pulp as an ingredient to impart flavor to Saison Ale-type beer. Moreover, it is a fruit that is still little explored in the industry, and besides imparting a striking flavor to the beverage, it can also nutritionally enrich it due to the presence of bioactive compounds, mainly antioxidants and organic acids. Thus, the objective of this work was to prepare beers by varying the concentrations of tamarind pulp and to evaluate their effect on physicochemical and sensorial characteristics, as recommended by Brazilian legislation, such as the identification and quantitation of their volatiles, bioactive compounds and sensory attributes, including the consumer acceptability index of the developed products.

## 2. Materials and Methods

### 2.1. Raw Materials

The following malts were used to produce the beers: Pilsen, Vienna, Chateau Melano 80 and Caramunich light, all from the Agraria (Entre Rios, Bahia, Brazil) brand. The hop varieties for bittering and aroma used in pellet form were of East Kent Goldings (4.1% α-acid, 2018 harvest-UK) and Saaz (2.9% α-acid, 2018 harvest-CZ), both from the Barth-Haas Group (Nürnberg, Germany). The brewer’s yeast used was freeze-dried Belle Saison from Lallemand (Montreal, QC, Canada). Ripe tamarind fruit (*Tamarindus indica* L.) was purchased at CEASA (Aracaju Supply Center; latitude: 10°54′34″ S 37°; longitude: 4′29″ W), during the 2019 harvest.

### 2.2. Chemicals

Sodium hydroxide, hydrochloric acid and sulfuric acid were purchased from Dinâmica (Indaiatuba, São Paulo, Brazil); 3,5-dinitrosalicylic acid (DNS) formic acid, ethyl acetate, acetonitrile and malic acid were obtained from Sigma-Aldrich (St. Louis, MO, USA); ethanol and methanol—both HPLC-grade—were obtained from Tedia High Purity Solvents (Rio Grande do Sul, Brazil). Acetone (HPLC grade) was obtained from J.T. Baker (Aparecida de Goiânia, Góias, Brazil). The chemicals were of HPLC grade or of analytical standard grade, and other chemical standards such as organic acids (acetic acid, butyric acid, fumaric acid, citric acid, L-ascorbic acid, malic acid, quinic acid, succinic acid and tartaric acid), sugars (fructose, glucose, nystose and sucrose), and phenolic compounds (acacetin, caffeic acid, chlorogenic acid, ferulic acid, gallic acid, *p*-coumaric acid, protocatechuic acid, vanillic acid, apigenin, biochanin A, catechin, chrysin, daidzein, epicatechin, ethyl gallate, kaempferol, luteolin, naringenin, rutin and vanillin) were obtained from Sigma-Aldrich (St. Louis, MO, USA).

### 2.3. Extraction of Tamarind Pulp

Firstly, the ripe fruits went through selection, followed by sanitization using sodium hypochlorite at a concentration of 200 ppm, and were then rinsed. After sanitization, the tamarind fruits were submerged in distilled water and left to rest for 2 h. This was carried out to facilitate the process of obtaining the pulp. The pulp was obtained mechanically using a pulper (Itametal, model Bonina—NB15, Itabira, Brazil) using a 1.5 mm diameter screen. The tamarind pulp was placed in polyethylene containers and subsequently frozen at the Laboratory of Flavor and Chromatographic Analysis (LAF) at the Federal University of Sergipe (UFS), São Cristóvão, Brazil.

### 2.4. Physicochemical Analyses of Tamarind Pulp

The pH was measured by using a Hanna Instrument, model HI 2221, Amorim, Portugal, total soluble solid content (TSS) were read using a BrasEq digital refractometer, Jarinu, Brazil. The other chemical analysis of tamarind pulp was carried out following the procedures outlined in the analytical techniques book published by the Instituto Adolfo Lutz [17], Association of Official Analytical Chemists [18] and BPVSG [19]. The values of the physicochemical properties of tamarind pulp are presented in Appendix A.1 (Table A1).

### 2.5. Beer Production

#### 2.5.1. Test Formulation

**Figure A1:**
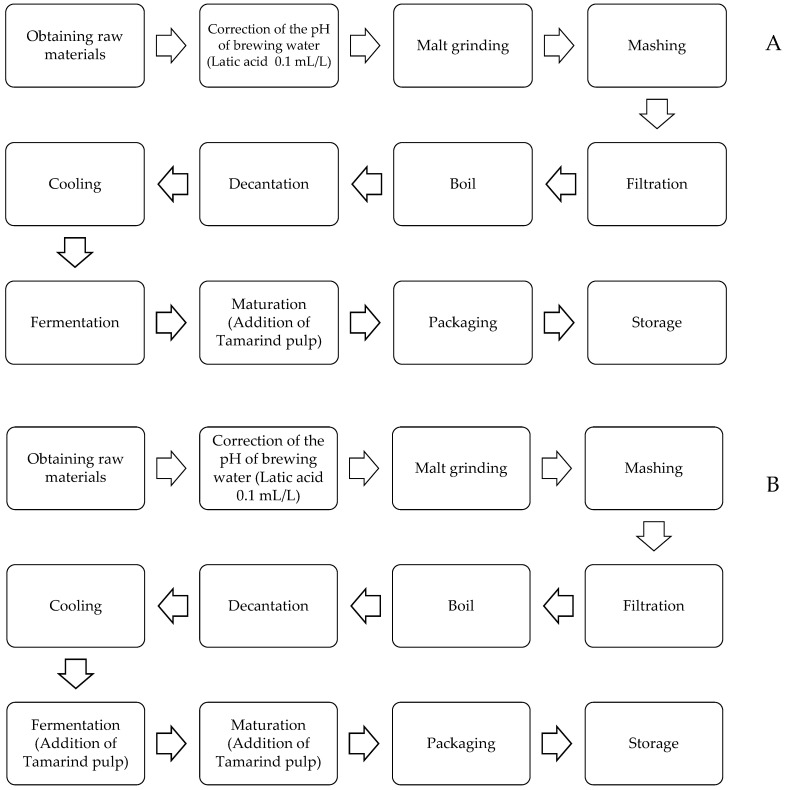
Flow diagrams during the production process of various beers. (**A**) Test formulation; (**B**) beer formulations.

The test beer formulation was prepared following the guidelines of the American Society of Brewing Chemists [20], designed to produce 20 L of final product. The malt composition was 3.72 kg Pilsen, 0.34 kg Vienna and 0.37 kg Chateau Melano 80. As regards the hops, the addition was of 35.69 g East Kent Goldings and 12.00 g Saaz, as bittering and aroma hops, respectively, and 11.00 g of freeze-dried Belle Saison yeast. Initially, tamarind pulp at a concentration of 15 g/L was tested, its addition being in the fermentation and/or maturation stages in order to evaluate the addition of the fruit pulp at these two stages. Figure A1 (Appendix A.3) shows the manufacturing process of Saison Ale craft beer with the addition of tamarind. This formulation was prepared for sensory evaluation by experts in craft beer and, consequently, for the production of subsequent formulations.

#### 2.5.2. Beer Formulations

The beers were prepared following the steps described in Section 2.5.1, to obtain 20 L of final product, with the following modifications: the malt composition was 6.65 kg of Pilsen, 0.61 kg of Vienna, and 0.50 kg of *Caramunich* light. The hops used were 65.32 g of East Kent Goldings and 12 g of Saaz, as bittering and aromatic hops, respectively, and 22 g of Belle Saison yeast. As for the addition of tamarind pulp, additions of 20, 40, and 60 g/L were tested. Figure A1 (Appendix A.3) shows the process of brewing Saison Ale craft beer with the addition of tamarind during the maturation phase.

### 2.6. Physicochemical Analysis and Characterization of Beer Samples

All the physicochemical analyses were carried out with decarbonated samples. To remove CO_2_, the samples were transferred to a 500 mL beaker and stirred with a glass rod, maintaining the beer temperature between 20 and 25 °C. Analyses were carried out in triplicate. The pH was measured by using a Hanna Instrument, model HI 2221; total titratable acidity (TTA), and total soluble solid content (TSS) were read using a BrasEq digital refractometer. The alcohol content and relative density were determined by using the methodology described by Instituto Adolfo Lutz [17]. Total reducing sugars (TRSs) were determined based on the colorimetric method of 3,5-dinitrosalicylic acid (DNS), described by the Embrapa [21]. The determination of bitterness through the International Bitterness Units (IBU) of the beers was carried out following the methodology described by the American Society of Brewing Chemists [20]. The color reading was measured at a wavelength of 430 nm according to the European Brewery Convention [22] protocol. The real extract value was obtained by converting the relative density value of the distillation residue for alcohol content analysis.

### 2.7. Identification and Quantification of Sugars and Phenolic Compounds of Beer by HPLC Systems

#### 2.7.1. Sugars

The identification and quantification of sugars were carried out following the methodology of Gomes [23] with slight modifications. Beer samples (10 mL) were centrifuged at 12,000 rpm for 15 min at 22 °C and filtered through a 0.45 µm PVDE filter for analysis by a high-performance liquid chromatography (HPLC) system (Shimadzu, Kyoto, Japan). The injection volume was 20 µL. The analysis was performed on an HPLC system, equipped with a pump (LC-20AT) and refractive index detector (RID-10A). The sugars were separated in a Supelcogel Ca column (300 mm × 7.8 mm, Supelco Inc., St. Louis, MO, USA), maintained at 80 °C. The elution was isocratic using deionized water mobile phase for 30 min, with a flow rate of 0.5 mL/min. To quantify glucose and fructose, calibration curves were constructed by analyzing a mixture of these sugars at different concentrations (1.0–10 g/L). The identification of the compounds was performed through the retention time of the peaks obtained in the chromatograms. The sugars sucrose, fructose and glucose were analyzed. The analysis of the samples was performed in triplicate.

#### 2.7.2. Phenolic Compounds

The quantification of phenolic compounds was carried out as described by Gomes [23] with slight modifications. Sample preparation was performed as described in Section 2.7.1. HPLC analysis was performed on a Shimadzu system (Kyoto, Japan) equipped with a quaternary pump (LC-20AT), a degasser (DGV-14A), and a manual sample injector (Rheodyne 7125 valve equipped with a 20 µL loop). Chromatographic separation was obtained using a Kinetex column (250 cm × 4.6 mm, 5 µm; Phenomenex, Torrance, CA, USA) and gradient elution with acidified pure water—1% acetic acid (A)—and acidified acetonitrile—1% acetic acid (B) mobile phase. The injection flow rate was 0.6 mL/min and the injected sample amount was 5 µL. Phenolic compounds were identified by comparing retention time with standards and quantification was performed by external standardization. The results were expressed in mg/L. Quantification was performed from a calibration curve using phenolic compound standards.

### 2.8. Identification and Quantification of Volatile Compounds in Beer Samples

The volatile compounds in beers were identified following the methodology of De Schutter [24] and adapted by Alvim [4]. Volatile compounds were extracted using the SPME (Solid Phase Microextraction) technique, where 10 mL of beer samples was placed in a 20 mL glass vial containing 3 g of NaCl. The sample remained in equilibrium with constant stirring at a temperature of 60 °C for 10 min. After the equilibrium time, the 50/30 mm of divinylbenzene-carboxene-polydimethylsiloxane (DVB/CAR/PDMS) fiber was exposed to the headspace, where extraction occurred at 60 °C for 30 min. After extraction, the SPME holder was placed in the gas chromatograph (GC) injector and the fiber was directly exposed to the hot injector inlet at 250 °C in splitless mode for 7 min.

The beer samples were analyzed in a gas chromatograph (GC, Agilent brand, model 7890A), coupled to a mass spectrometer (MS) (Triple Axis Detector MSD Agilent 7000A). A DB5-MS capillary column (30 m × 0.25 mm i.d. × 0.25 mm film thickness) was used for separation. The flow rate of the ultra-high purity (99.9999%) helium carrier gas was maintained at 1 mL/min at a constant flow. The column temperature was maintained at 40 °C for 2 min and increased to 250 °C at 10 °C/min, with a final retention time of 2 min. The ion source temperature was set to 280 °C and the electron impact mode was at 70 eV. Detection was performed in scan mode with the mass/charge ratio varying between 30 and 400 *m*/*z*.

Volatile compounds were tentatively or positively identified by comparing the mass spectra of the sample compounds with that of the NIST (National Institute of Standards and Technology) database; comparing the linear retention index (LRI) of the standards, which was calculated by injecting a series of alkanes (C7–C30) under identical analytical conditions; and comparing with those of the scientific literature and other online databases, viz., Flavornet, Pherobase and PubChem. Quantification of volatile compounds in beer samples was carried out by internal standardization, using 1-octanol (Sigma-Aldrich, São Paulo, Brazil) as the internal standard, and the results were expressed as concentration (mg/L ± standard deviation).

### 2.9. Sensory Evaluation of Beer Samples

The sensory analyses in this present work were carried out with approval from the ethics committee under process No. 3.214.563, published by the UFS Research Ethics Committee.

#### 2.9.1. Sensory Evaluation of the Test Formulation

In order to obtain better responses regarding the sensory parameters evaluated, aiming at improving the next formulation, the sensory analysis of the formulation was carried out with 10 master brewer’s panelists, adults over 18 years old and under 60 years, of which 8 were men aged between 22 and 53 years and 2 were women aged between 37 and 46 years, recruited on a completely voluntary basis. These panelists were either consumers or connoisseurs of craft beer and have knowledge of its manufacturing process. The first sensory evaluation aimed to assess the best addition of tamarind pulp in the formulation, at either the fermentation or maturation stage.

A new-point structured hedonic scale test was applied, anchored by scores varying from 1 (extremely disliked) to 9 (extremely liked), and the following attributes were evaluated: appearance, color, aroma, turbidity, flavor and overall assessment. To assess purchase intention, a five-point scale was used, which ranged from 1 (certainly would not buy) to 5 (certainly would buy). The acceptance index of beer containing tamarind pulp was also calculated using the equation IA (%) = A×100/B, where A = average score obtained for the product and B = maximum score given to the product. An acceptability index with good repercussion was considered as ≥70%.

#### 2.9.2. Sensory Evaluation of the Beer Formulations

The sensory analysis was conducted in a room with individual cabins, which were odorless and had white light, on the premises of the Sensory Analysis Laboratory of the Department of Food Technology (DTA) of the Federal University of Sergipe (UFS). There were a total of 62 untrained tasters, all, adults over 18 and under 60 years of age; among these, 38 were men, aged between 18 and 43 years, and 24 were women, aged between 18 and 46 years, recruited on a completely voluntary basis. The sensory evaluation sought to evaluate the beers to which three concentrations (20 g/L; 40 g/L and 60 g/L) of tamarind pulp were added in the maturation stage during the beer production process. The same sensory tests described in Section 2.9.1 were applied and the same sensory attributes were evaluated.

### 2.10. Statistical Analysis

The tamarind and beer characterization analyses were assessed by using Analysis of Variance (ANOVA), and the significance of the models was confirmed by the F test. The means in significant models were compared using the Tukey test at a significance threshold of 5% with SAS software version 9.4.

## 3. Results and Discussion

### 3.1. Sensory Evaluation of the Test Formulation

The first sensory analysis aimed to determine the most appropriate stage for adding tamarind pulp (fermentation or maturation). The panel consisted of master brewer adults (18–60 years) familiar with craft beer production. The results of the sensory evaluation and purchase intention are shown in Table 1.

ANOVA revealed no significant differences (*p* ≤ 0.05) among samples for any sensory attribute, likely due to the low concentration of tamarind. Mean scores for aroma, flavor, and overall impression ranged from “slightly liked” (6) to “slightly disliked” (4), while other attributes achieved “moderately liked” (~7). Judges reported that aroma and flavor lacked the expected fruity and citrus notes, attributing this to fermentation and maturation conditions. The highest scores for these attributes were obtained for BTDM (addition during maturation) (Table 1).

Among other characteristics, color, turbidity, carbonation, and foam stability received the highest ratings. BTDM showed greater turbidity, probably due to pulp residues, while the foam of all beers was well-formed and persistent. Judges suggested replacing Chateau Melano 80 with a lighter malt to better align color with the Saison style.

**Figure A2:**
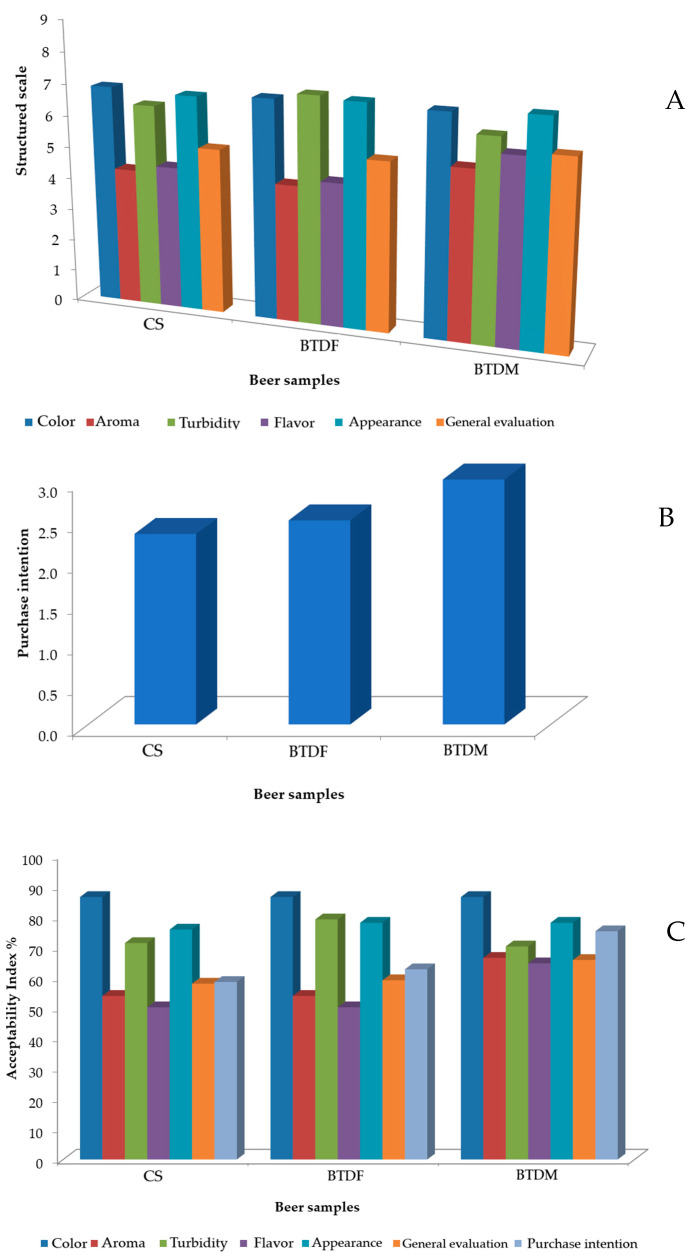
Average scores obtained through sensory analysis using a structured hedonic scale (**A**), purchase intention (**B**) and acceptability index (**C**) of the beer samples.

Beers with tamarind pulp achieved approval rates of ≥70% in terms of color, turbidity and appearance. In contrast, flavor, aroma and overall evaluation were below this threshold. However, BTDM (tamarind added during maturation) achieved the highest values for these attributes, all of which were ≥60%, surpassing CS and BTDF (see Appendix A.4, Figure A2).

BTDM obtained the highest average score on the hedonic scale and the highest acceptability index among the samples, with 75% of participants indicating purchase intent compared to 62.5% for BTDF and 58.3% for CS. Overall, adding tamarind pulp during maturation showed the greatest potential for optimizing the less expressive sensory attributes.

The results obtained through sensory analysis performed with the test of beer formulation enabled the selection of the best stage between fermentation and maturation for the addition of tamarind, as well as providing a highly significant field of study for the continuity of the experiment. As master brewers and craft beer consumers, the panelists of the test formulation provided a more detailed assessment of the product in question. They offered specific comments on the production parameters and ingredients used, which could enable improvements to be made in the quality of the final product.

The first suggestion from the panelists was to change one of the malts used in production so that it could bring a color more characteristic of the Saison style to the finished product. Thus, *Chateau Melano 80* malt was replaced by *Caramunich light*. Regarding the addition of tamarind pulp, the panelists pointed out that the fruit was not perceptible to the senses. Thus, the addition, which in the first formulation was 15 g/L, was increased to 20, 40, and 60 g/L, these three being chosen because the second stage of this study aimed to perceive the contribution of different concentrations of tamarind pulp. Based on the data obtained from sensory analysis and suggestions from expert panelists, the beer formulations were prepared as described in Section 2.5.

### 3.2. Characterization of Beer Formulations

The results of the physicochemical analysis and characterization of the Saison-type craft beer made by the addition of fresh tamarind pulp in three different concentrations (20, 40 and 60 g/L) are presented in Table 2. Unlike the first formulation, some physicochemical parameters (pH, TSS and TTA) were monitored during some stages of the brewing process until the final product was obtained. These stages were as follows: start of mashing (SM); increase in temperature during mashing to 70 °C (M 70.) and at 78 °C (M 78.); beginning of boiling (BB); end of boiling (EB); start of fermentation (SF); days of fermentation (F1st day; F2nd day; F3rd day; F5th day; F6th day and F7th day); on the seventh day of maturation (M7th day); packaging (P); and final beer (RB). It is worth mentioning that until the beginning of fermentation, all values were unique. During the fermentation process, the must was divided into four fermenting buckets so as to study the incorporation of tamarind pulp, while having the control bucket, that is, the one without the addition of tamarind pulp (CS), and the buckets with the addition of tamarind pulp of 20, 40 and 60 g/L in the maturation stage (BTDM20 g/L, BTDM 40 g/L and BTDM 60 g/L, respectively).

**Figure 1:**
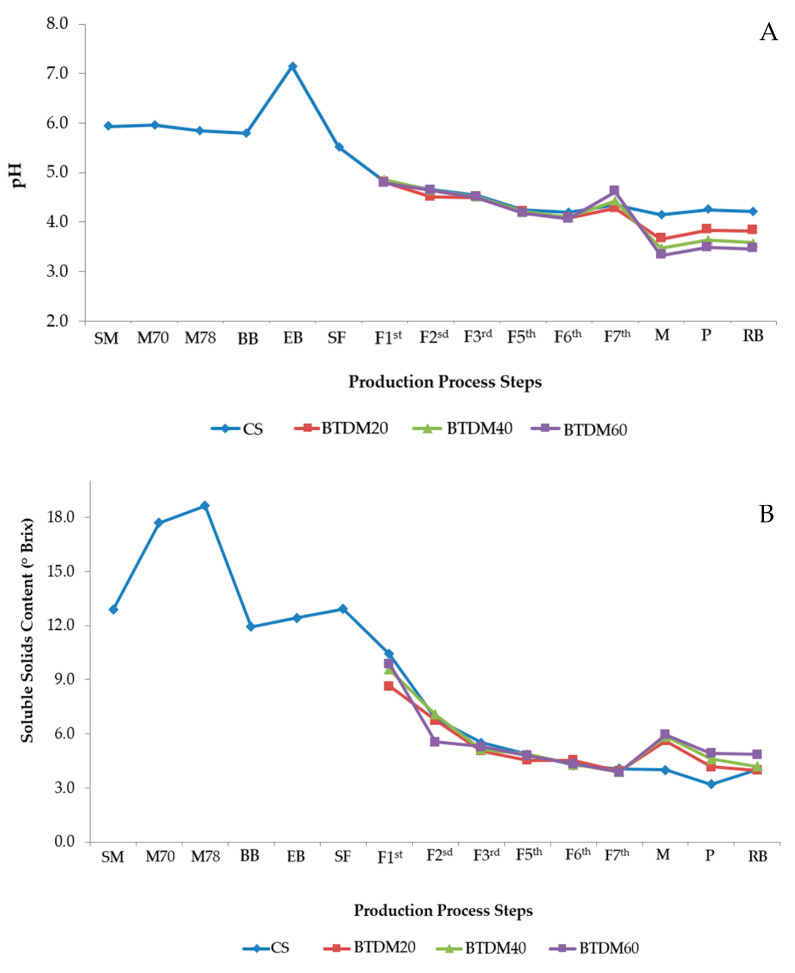
pH variations and total soluble solid contents during various operations of the beer production. (**A**) Evaluation of pH during some stages of the brewing process until the final product was obtained; (**B**) evaluation of °Brix during some stages of the brewing process until the final product was obtained.

The pH in the initial stages of beer production showed values close to 6 with a slight drop. This value can be attributed to the pH value of the mineral water used in the mashing. At the end of boiling, the pH presented its highest value (7.14) during the entire monitoring period. This may have occurred due to the concentration of compounds in both the malt and hops added at this stage. At the beginning of fermentation, the must had a pH value of 5.51; this value decreased over the period of fermentation where all samples presented values close to 4 (Figure 1A). The decrease in pH during the fermentation process is attributed to the increase in CO_2_ concentration, as well as the production of organic acids, during the anaerobic phase of yeast metabolism [25]. It is important to highlight that the drop in pH values contributes to the quality of the product, as values below 4.40 prevent the development of contaminants, such as microorganisms of the *Enterobacter* and *Citribacter genera*.

At the start of mashing (SM), the soluble solid content was of 12.87 °Brix; with the mashing temperature ramp, the soluble solids increased, and the values presented were of 17.68 and 18.63 °Brix for 70 °C and 78 °C, respectively (Figure 1B). This is due to the fact that the polysaccharides (starch) present in the malt underwent hydrolysis after heating, thus increasing the monosaccharaides (glucose) value.

After mashing, the soluble solid values decreased due to the increase in water during filtration, which caused the dilution of these solids. On the first day of fermentation, the soluble solid content presented a value close to 10 °Brix for all samples and these values decreased throughout the fermentation process. This was expected as the °Brix value is related to the concentration of fermentable sugars available in the fermentation process. In the must solution and as the days of fermentation progress, the yeast uses these matrices for its growth/development and for the conversion into alcohol and CO_2_. On the seventh day of fermentation, the BTDM 20, 40 and 60 g/L samples presented values of 5.61, 5.85 and 5.95 °Brix, respectively; these values can be attributed to the addition of tamarind pulp. The finished beers had °Brix values close to 4, and all samples differed statistically (*p* ≤ 0.05) from each other, which can be seen in Figure 1B.

**Figure 2:**
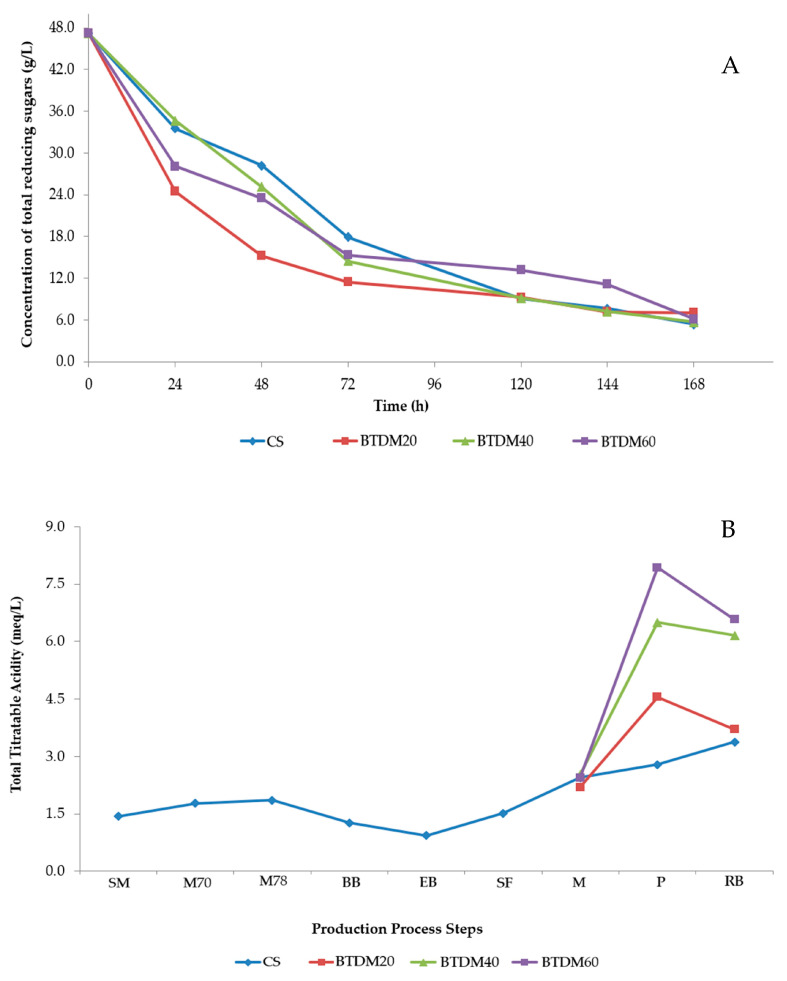
Monitoring of total reducing sugars and total titratable acidity during beer production. (**A**) Total reducing sugars monitored as a function of fermentation time (hours); (**B**) total titratable acidity evaluated at discrete stages of the brewing process.

Monitoring of TRS was carried out over seven days of fermentation, and the results can be seen in Figure 2A. It was observed that the concentration of TRS decreased over the days of fermentation, varying from 141 g/L (time 0 h) up to concentrations between 16 and 21 g/L (time 168 h). The decrease in this parameter is due to the use of these components by yeasts as a substrate for their development/growth, as well as for the biosynthesis of alcohol and CO_2_. The inclusion of tamarind pulp in the maturation process may have influenced this parameter, since even this is considered a second stage of fermentation; during maturation. The yeast no longer presents high activity in its metabolic syntheses; therefore, the fermentable components that could be used in tamarind pulp may not have been assimilated by it.

As mentioned previously, Brazilian legislation does not have a standard value for total acidity in beers; therefore, this analysis is only considered to characterize the drink based on its acidity. It is possible to observe in Figure 2B that the acidity levels during the process varied; this can be attributed to the insertion of inputs such as hops during boiling, as well as to the addition of water in the filtration stage. The inclusion of tamarind pulp in the maturation phase influenced the acidity of the samples, as well as in the final product, imparting an acidic flavor and aroma to the beers. The final beers had average acidity values that differed between the samples (*p* ≤ 0.05). However, the BTDM 60 g/L sample showed higher acidity, as can be seen in Figure 2B. It is important to highlight that the acidity of beer increases with its carbonation, as carbonic acid increases the acidity of the drink.

Regarding the alcohol content of beers, the BTDM 60 g/L sample presented the highest value, which was 7.6% (Table 2). Regarding the color of the beers, all beers are classified as dark according to legislation, as they obtained values greater than or equal to 20 EBC units.

In relation to the real extracts of the samples, (Table 2), an increase in this parameter can be observed as the pulp concentration increases. Given the values obtained, in accordance with Brazilian Legislation, the samples were classified as beers with medium extract content. Among the beers studied, the sample with the addition of 60 g/L of tamarind pulp presented higher °Brix and real extract values. The addition of tamarind pulp may have influenced these factors, as its insertion occurred during the maturation phase. Even though it is considered a second fermentation, the kinetic activity of the yeast has already decreased and may even have ceased. Thus, many fermentable components present in tamarind were not converted into alcohol and the percentage of real extract indicates the amount of ingredients not transformed into alcohol that are found in beer after fermentation.

Considering the Beer Judge Certification Program (BJCP) and the parameters listed for classifying beer as Saison, the beers obtained may fall into this category. The original gravity (OG) of the wort was 1.052, but the final gravity was 1.015, almost close to the standard value defined by the BJCP. In the case of bitterness, the beers are within the 20–35 IBU classification range, as they all presented values between 31 and 29 IBU. Finally, in relation to chlorination, the BJCP classification unit is the Standard Reference Method (SRM), converting the values obtained in the EBC to SRM. In relation to chlorination, the BJCP classification unit is SRM, leading to the conversion of values which were obtained in EBC for SRM; all beers were classified as clear, with values of 11.76, 11.86, 9.76 and 10.08 SRM for samples CS, BTDM 20, 40 and 60 g/L, respectively.

### 3.3. Identification and Quantification of Bioactive Compounds and Sugars of Second Beer Formulations by High-Performance Liquid Chromatography (HPLC)

#### 3.3.1. Sugars

Monitoring sugar concentration during the beer production process is very important. The carbohydrates present in the malt grain must be hydrolyzed during the mashing process so that they can be assimilated by yeast during fermentation, thus causing the production of ethanol and other compounds that influence the organoleptic characteristics of this product. Thus, identification and quantification of sugars in some stages of beer production are important, the results of which are presented in Appendix A.2 (Table A2).

In the maturation stage, the beers were analyzed in four samples. Sucrose and glucose were identified; the two sugars for the samples without the addition of tamarind pulp (CS) and with 20 g/L of pulp (BTDM 20 g/L) did not differ statistically (*p* < 0.05) from each other, but they differed from the beers obtained with 40 and 60 g/L of tamarind pulp. However, these two beers did not differ from each other in their sucrose contents. In relation to glucose at maturation, the BTDM 60 g/L sample exceeded the 5% significance level of the other beers. During packaging, glucose was found to increase in all beers during the maturation stage. CS and BTDM 20 g/L beers did not differ from each other at 5% significance, but differed from the two others, BTDM 40 and BTDM 60. The increase in glucose concentration and identification of fructose occurs because invert sugar is formed at this stage (sucrose heated in water, causing the disaccharide to break down into glucose and fructose) to carbonate the drink in the bottle.

Martínez [26] investigated the effect of adding different concentrations of persimmon juice at the start of beer fermentation, focusing on the identification and quantification of sugars throughout the process. The authors observed that glucose and maltose levels decreased as fermentation progressed, while fructose—the most abundant fermentable sugar in persimmon juice—remained at higher concentrations in beers produced with greater amounts of the fruit.

#### 3.3.2. Phenolic Compounds

The data on phenolic compounds found by HPLC systems in beers are tabulated in Table 3. Five phenolic acids and three flavonoids were identified and quantified. Artepelin C, among the phenolic acids, was the one that presented the highest concentration in all samples, where all samples would differ statistically at 5% significance. In relation to the other phenolic acids identified, 2-hydroxycinnamic acid was in the lowest amount, but there was an increase in it directly related to the concentration of the addition of tamarind pulp, as also observed for ascorbic acid, being identified only in samples without addition of tamarind pulp (CS) and with addition of pulp (BTDM 20 and 40 g/L). Caffeic acid was only identified in samples with 20 and 40 g/L of tamarind pulp, but there was no statistical difference (*p* < 0.05) between these beers. Finally, the gallic acid identified in beers with the addition of tamarind pulp had its concentration reduced when the fruit pulp was added, with BTDM 20 g/L being the beer that presented the highest concentration (0.5422 mg/L).

In relation to the three flavonoids identified and quantified, the sample without the addition of tamarind was the only one that did not present concentrations of catechin, while the beers with tamarind addition, even differing from each other at 5% significance, presented similar values for this phenolic compound. The samples with the addition of 20 and 40 g/L of tamarind pulp were those that presented the highest amounts of epicatechin, and both differed from the other two samples. Ethyl gallate was the only phenol that, when the addition of tamarind pulp increased, had its values increased. Thus, the sample with 60 g/L of tamarind pulp was the one that exhibited the highest amount (0.1116 mg/L) of it.

It is known that the raw materials used in beer production, as well as the parameters of malt processing and brewing, affect the chemical composition of the final product, which may explain the divergences in the profile of phenolic compounds between the beers analyzed in the present study. However, regardless of its profile, it is known that high levels of phenolic compounds improve the flavor and stability of beer [27].

### 3.4. Identification and Quantification of Volatile Compounds of Beer Formulations

The evaluation of the volatiles’ composition and aromas of beers makes it possible to identify variations in the process that lead to the emergence of strange and undesirable aromas, which are considered critical issues for production of beers. Beers considered to be of artisanal production require greater attention when it comes to the development of aromas, as it is a process in which pasteurization and filtration processes do not affect, and these tend to help preserve the beer by preventing its aromatic and flavor profile from being influenced during storage and aging [28].

The volatile compounds of beers without and with the addition of tamarind pulp during maturation are presented in Table 4. Twenty-seven compounds were identified in the samples. 3-methyl-1-butanol (isoamyl alcohol) and phenylethyl alcohol showed greater expressiveness within the alcohol class. The main products of fermentation carried out in beer by yeast produce alcohols which contribute to the construction of the aroma in beer. The compounds formed in this process improve the quality of the beer, contributing not only to the flavors and aromas, but also to the amount of nutrients in this drink [29].

Among the carboxylic compounds present in beers are aldehydes. The aldehydes that significantly influence the flavor of beers originate from the Strecker degradation of amino acids, the Maillard reaction and the oxidation of fatty acids [30]. Carboxylic acids are compounds that influence the flavor of beers; short-chain acids contribute to reducing pH during fermentation, giving the product a sour flavor [31]. Among the class of aldehydes, the compound that stood out for having the highest percentage of area was benzaldehyde, with sample BTDM60 having the highest value (34.23 mg/L).

Esters are characterized by pleasant fruity odors that contribute to the aromatic delicacy of the beer. Esters are the most important chemical class in the volatile’s composition of beers, and there was an increase in these compounds with the inclusion of increased tamarind pulp. These compounds are formed during maturation, a step that aims to improve the odor and flavor of the drink, with esters being responsible for these characteristics in addition to helping to maintain the stability of the product, preventing oxidation from occurring [32].

Among the esters, ethyl octanoate and phenylethyl acetate were found in the highest concentrations, conferring fruity and floral aromatic notes, respectively. The other esters in this group were found in almost all the samples and are attributed to fruity aromas such as banana and apple. Among the group of esters identified in the samples, phenylethyl acetate had the highest concentration, varying from 20.35 (CS) to 27.83 mg/L (BTDM60). Evaluating these results with the sensory analysis, the tasters described the samples with added tamarind as having fruity and citrus aromas.

More than 90 esters have been identified in beers, but the most prominent in terms of volatile and aromatic characteristics were octanoate acetate, ethyl isoamyl acetate, ethyl acetate, decanoate acetate and phenylethyl acetate [33]; except for ethyl acetate, all other compounds were identified in the beers analyzed in this study. Viejo [28] aimed to study some beers with 3 types of fermentation, 10 of which were top-fermented (Ale), including one in the Saison style, the style of the beer developed in the present study. The author and his collaborators identified that these beers had fruity aromas of apple, pineapple and floral aromas such as rose and honey, assigning them the chemical class of esters.

The vast majority of compounds in the terpene class are derived from hops [5]; within this class, three compounds were identified in the samples. Citronellol was the terpene/monoterpene alcohol found in all samples; it has a characteristic floral aroma of rose, with the highest percentage (24.75 mg/L) present in the BTDM60 sample. Linalool, which has a floral and citrus odor, was identified in the samples without added tamarind (CS) and with addition of 40 g/L (BTDM40) of tamarind pulp.

Da Silva [9] investigated the influence of mandacaru pulp, added as an adjunct to beers at different concentrations, on volatile compounds and identified sixty-one volatile compounds, among which the most prominent were phenethyl alcohol and phenethyl acetate ester, compounds that were also identified in the beer samples evaluated in this study. Yang [34] developed a new larger beer with jujube as an adjunct and when evaluating the volatiles profile of these beers they reported that the ester class was the most abundant with a total of 26 compounds identified, followed by the alcohol class. Among the compounds identified were 3-methyl-1-pentanol, phenethyl alcohol, isoamyl acetate and ethyl octanoate, all of which were present in the beers with tamarind pulp. The volatile composition of the beers developed with tamarind pulp showed a similar volatile composition to the results reported in the literature.

### 3.5. Sensory Evaluation of Beer Formulations

Sensory analysis was performed by 62 panelists, including 38 men, aged between 18 and 43 years, and 24 women, aged between 18 and 46 years. The evaluation form included two questions regarding beer consumption; 53 panelists (34 men and 19 women) usually consume commercial beers but only 9 (4 men and 5 women) did not have this habit. Regarding the consumption of craft beers, 48 of the panelists (31 men and 17 women) stated that they consume or have consumed some type of craft beer, while 14 (7 men and 7 women) have never tasted this type of beer. These questions were raised with the aim of understanding the public to which the product was offered, because unlike the first sensory evaluation where the tasters knew, consume and even produce craft beers, this second sensory evaluation sought to evaluate the behavior of the beers in front of a wider public, who do not have the habit of consuming craft beers, and this could become a new product for these consumers as well as others.

The results of the sensory analysis and purchase intention of the four beer formulations are presented in Table 5. When performing the analysis of variance (ANOVA) for the attributes, it can be observed that there was no significant difference (*p* ≤ 0.05) between the samples in any of the sensory parameters evaluated. Among the attributes, almost all presented averages on the hedonic scale between “I liked it moderately” (7) and “I liked it slightly” (6), with the exception of the flavor attribute for the samples BTDM 40 g/L and BTDM 60 g/L.

According to the panelists, the samples with the addition of 40 and 60 g/L of tamarind pulp were the least pleasing in terms of flavor, presenting high residual acidity, and a bitter and strong taste; some tasters raised the issue of the dry taste, which could also be attributed to the high acidity of the samples, but these samples were characterized as having a refreshing characteristic. For the beer with 20 g/L of tamarind pulp, acidic characteristics were also attributed to the flavor, although milder when compared to the previous two beers. In relation to the flavor, the fruity, pleasant and refreshing notes were the predominant ones for this sample, as well as pleasant and citrus/fruity aroma. Finally, as regards the control beer, in which there was no addition of tamarind pulp (CS), the judges highlighted that it was close to commercial beers, being less bitter, with a pleasant and smooth taste.

**Figure 3:**
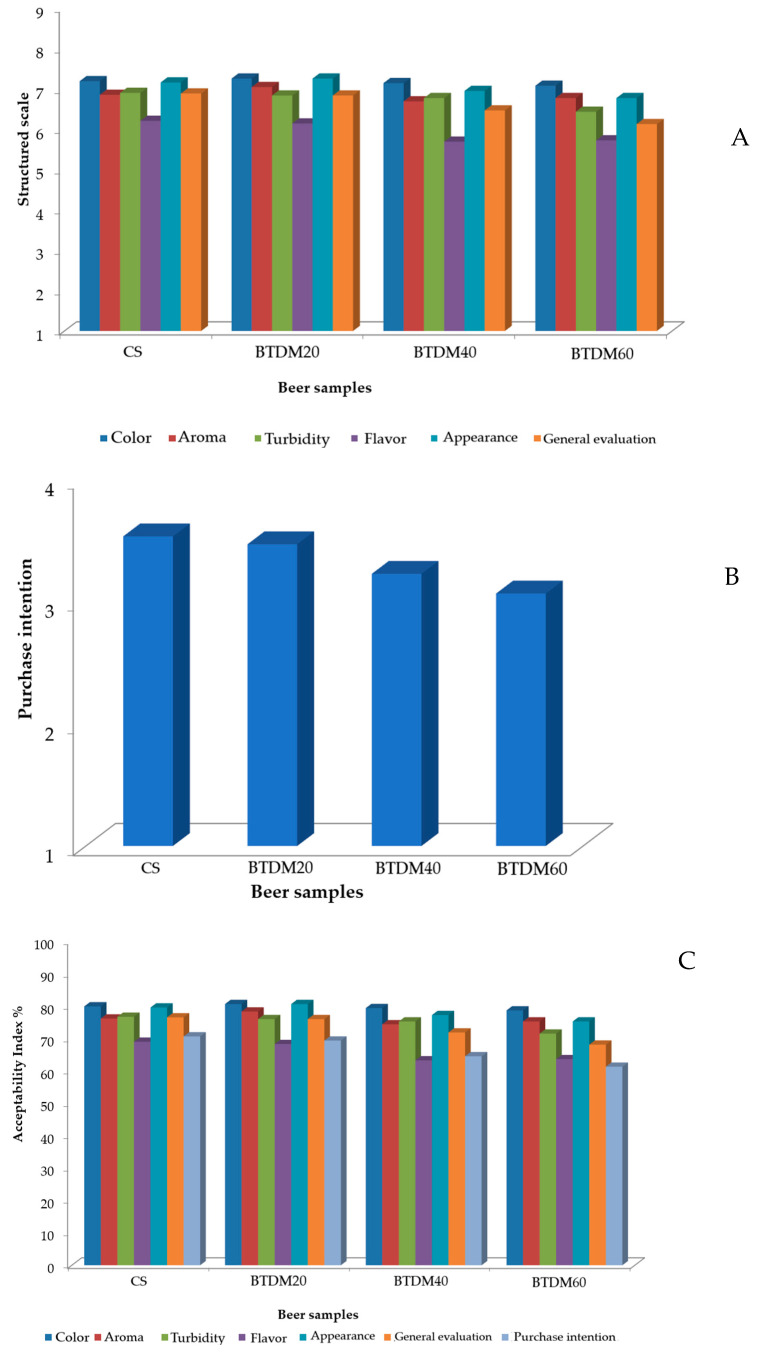
Average scores obtained through sensory analysis using a (**A**) structured hedonic scale for the evaluated parameters, (**B**) for purchase intention, and (**C**) acceptability index.

The distribution of the average scores obtained in the sensory evaluation is shown in Figure 3. Although no statistically significant differences were observed among the samples and the mean attribute values did not vary markedly, the CS sample (without tamarind) and the BTDM 20 g/L sample (20 g/L of tamarind pulp added during maturation) received slightly higher scores than the other formulations (BTDM 40 g/L and BTDM 60 g/L), particularly for flavor. Purchase intention also showed no statistical differences among the samples (*p* < 0.05). It was observed that a small decline in the notes was directly proportional to the amount of tamarind pulp addition; all the notes were framed in ‘Maybe I would buy/maybe I wouldn’t buy” (3) (Figure 3B).

In relation to the acceptability index, the beers with added tamarind presented values above 70%. Evaluating globally, all beers had good acceptability rates. The only attribute where all samples presented values below 70% was flavor; however, the CS and BTDM 20 g/L samples presented values close to the proposed hypothesis, with 69 and 68.28%, respectively. Regarding the overall evaluation, the sample with 60 g/L of tamarind pulp (BTDM 60 g/L) was the only one that did not reach 70%. Finally, regarding purchase intention, a decrease in the value of the acceptability index was observed with the increase in the addition of tamarind pulp again, where only the sample without the addition of tamarind pulp (CS) presented a value equal to the hypothesis proposal, followed by BTDM 20 g/L, BTDM 40 g/L and BTDM 60 g/L, which obtained 69%, 64% and 61%, respectively (Figure 3C).

The results of the sensory evaluation indicate that, although no statistically significant differences were observed between the formulations, the acceptance of the beers was influenced by the level of tamarind addition. The highest concentrations (40 and 60 g/L) intensified the acidity and bitterness, reducing the flavor notes, overall acceptance, and purchase intention. In contrast, the formulation with 20 g/L presented a better sensory balance, with fruity and refreshing notes that favored its acceptance, while the control sample maintained a profile closer to that of commercial beers.

## 4. Conclusions

This study demonstrates that incorporating tamarind pulp as an adjuvant in Saison-type craft beer is technologically feasible without compromising fermentation performance. The addition of different concentrations of pulp (20–60 g/L) during the maturation stage did not compromise the physicochemical parameters, including bitterness (29–31 IBU), color (19–23 EBC), and extract (3.3–2.5 g extract in 100 g of solution), which remained within acceptable limits, confirming the stability of the process. In addition, the alcohol content was not affected, with final values varying between 6.1% and 7.6% (*v*/*v*), which is an adequate range for the style of beer produced.

Volatile component analysis revealed that the addition of tamarind during maturation promoted a clear enrichment of active aromatic compounds, particularly esters. The beer produced with 20 g/L of tamarind pulp (BTDM20) had the most balanced volatile profile, with notable increases in the main fruity and floral esters, such as isoamyl acetate, ethyl octanoate, and phenethyl acetate, compared to the control. On the other hand, although the higher amounts of tamarind pulp (40 and 60 g/L) presented slightly higher concentrations of these compounds, acidity and bitterness were affected, as can be seen in the physicochemical analyses (Table 2), resulting in a decrease in sensory benefits.

In addition to the effects on volatile composition, tamarind supplementation significantly influenced the phenolic profile of the beers. Beers containing tamarind pulp had higher levels of phenolic compounds such as gallic acid and catechin, with the highest concentrations found in samples BTDM20 and BTDM40, at 0.54 and 0.24 mg/L, respectively. Furthermore, these compounds were not detected in the control sample (CS), confirming the fruit as an effective source of bioactive compounds.

Sensory evaluation corroborated the chemical data, as BTDM20 achieved the highest overall acceptance, with acceptance indices close to or above the 70% threshold commonly associated with consumer acceptance, as well as higher purchase intention compared to beers containing 40 and 60 g/L of tamarind pulp. These results indicate that the addition of 20 g/L of tamarind pulp during maturation represents the optimal condition, providing enhanced aroma complexity and consumer acceptance while preserving physicochemical quality, and supporting tamarind’s potential as an innovative ingredient for fruit-based craft beers.

## Figures and Tables

**Table 1:** Average values of scores given by panelists for sensory attributes of test formulations.

Attributes	Control (CS)	Beer with Tamarind Pulp Added in the Fermentation (BTDF)	Beer with Tamarind Pulp Added During Maturation (BTDM)
Color	6.9 ± 0.99 ^a^	6.9 ± 1.10 ^a^	6.9 ± 1.20 ^a^
Aroma	4.3 ± 2.00 ^a^	4.3 ± 1.82 ^a^	5.3 ± 2.31 ^a^
Turbidity	6.4 ± 1.71 ^a^	7.1 ± 1.10 ^a^	6.3 ± 2.16 ^a^
Flavor	4.5 ± 2.01 ^a^	4.5 ± 2.01 ^a^	5.8 ± 2.15 ^a^
Appearance	6.8 ± 1.22 ^a^	7.0 ± 1.24 ^a^	7.0 ± 1.41 ^a^
General evaluation	5.2 ± 1.68 ^a^	5.3 ± 1.63 ^a^	5.9 ± 1.91 ^a^
Purchase intention	2.3 ± 1.50 ^a^	2.5 ± 1.43 ^a^	3.0 ± 1.41 ^a^

Results presented as average values. Equal letters on the same line indicate that the means do not differ statistically at *p* < 0.05 using the Tukey test.

**Table 2:** Physicochemical characterization of control and tamarind-added beers during the maturation stage.

		Beers		
Parameters	Control (CS)	Beers with 20 g/L of Tamarind Pulp Added During Maturation (BTDM20)	Beers with 40 g/L of Tamarind Pulp Added During Maturation (BTDM40)	Beers with 60 g/L of Tamarind Pulp Added During Maturation (BTDM60)
Alcohol content % (*v*/*v*)	6.10 ± 0.01 ^c^	7.10 ± 0.03 ^b^	7.10 ± 0.04 ^b^	7.60 ± 0.01 ^a^
Bitterness (IBU)	31.66 ± 0.01 ^a^	30.00 ± 0.04 ^b^	29.02 ± 0.03 ^c^	29.60 ± 0.03 ^d^
Color (EBC)	23.17 ± 0.06 ^a^	23.37 ± 0.11 ^b^	19.24 ± 0.07 ^d^	19. 84 ± 0.04 ^c^
Real extract % (g extract in 100 g of solution)	2.50 ± 0.01 ^d^	2.75 ± 0.03 ^c^	3.35 ± 0.00 ^a^	3.30 ± 0.07 ^b^

Results presented are the average values. Equal letters on the same line indicate that the means do not differ statistically at *p* < 0.05 using the Tukey test.

**Table 3:** Phenolic compounds of control and tamarind-added beers during the maturation stage.

		Beers		
Phenolic Compounds (mg/L of Beer)	Control (CS)	Beers with 20 g/L of Tamarind Pulp Added During Maturation (BTDM20)	Beers with 40 g/L of Tamarind Pulp Added During Maturation (BTDM40)	Beers with 60 g/L of Tamarind Pulp Added During Maturation (BTDM60)
Ascorbic acid	0.43 ± 0.02 ^b^	0.53 ± 0.00 ^a^	0.70 ± 0.01 ^c^	NQ
Caffeic acid	NQ	0.03 ± 0.00 ^a^	0.03 ± 0.00 ^a^	NQ
Gallic acid	NQ	0.54 ± 0.00 ^a^	0.47 ± 0.00 ^b^	0.46 ± 0.01 ^c^
2-hydroxycinnamic acid	0.02 ± 0.00 ^b^	0.02 ± 0.00 ^c^	0.02 ± 0.00 ^b^	0.02 ± 0.00 ^a^
Artepelin C	4.31 ± 0.27 ^b^	4.22 ± 0.44 ^d^	4.29 ± 0.04 ^c^	4.48 ± 0.32 ^a^
Catechin	NQ	0.23 ± 0.00 ^c^	0.24 ± 0.00 ^a^	0.23 ± 0.01 ^b^
Epicatechin	0.24 ± 0.01 ^c^	0.28 ± 0.01 ^a^	0.27 ± 0.00 ^b^	0.20 ± 0.09 ^d^
Ethyl gallate	0.10 ± 0.00 ^d^	0.10 ± 0.01 ^c^	0.10 ± 0.01 ^b^	0.11 ± 0.00 ^a^

Results presented as average values. Equal letters on the same line indicate that the means do not differ statistically at *p* < 0.05 using the Tukey test; NQ (not quantified).

**Table 4:** Volatile compounds identified and quantified in beers without tamarind pulp (CS) and with tamarind pulp added during maturation (BTDM 20, 40, and 60 g/L), expressed as concentration (mg/L ± standard deviation).

N.	Compounds	LRIa	LRIb	Control (CS)	Beers with 20 g/L of Tamarind Pulp Added During Maturation BTDM20	Beers with 40 g/L of Tamarind Pulp Added During Maturation BTDM40	Beers with 60 g/L of Tamarind Pulp Added During Maturation BTDM60	Odor
**Organic acids**								
**1**	Hexanoic acid	1013	1009	1.87 ± 0.20	11.31 ± 0.74	13.24 ± 0.18	NQ	Fruity
**2**	Octanoic acid	1170	1180	42.39 ± 2.31	2.31 ± 0.25	NQ	NQ	Fatty
**3**	Dodecanoic acid	1572	1569	1.50 ± 0.03	1.91 ± 0.21	1.98 ± 0.06	2.51 ± 0.15	Coconut oil
**4**	Decanoic acid	1366	1382	2.46 ± 0.27	3.08 ± 0.34	4.36 ± 0.12	4.51 ± 0.49	Citric
**Alcohols**								
**5**	3-methyl-1-butanol	741	739	4.51 ± 0.49	16.06 ± 1.05	17.97 ± 1.18	18.77 ± 1.02	Fruity
**6**	Phenethyl alcohol	1109	1117	2.16 ± 0.24	24.20 ± 1.32	13.73 ± 0.90	17.47 ± 1.14	Honey, rose
**7**	1-Dodecanol	1470	1471	0.48 ± 0.08	0.37 ± 0.06	0.55 ± 0.06	NQ	NQ
**Aldehydes**								
**8**	(*E*)-2-nonenal	1165	1159	1.78 ± 0.27	NQ	NQ	NQ	Green
**9**	2-Undecenal	1369	1363	14.21 ± 0.93	NQ	NQ	NQ	Fruity
**10**	Dodecanal	1416	1407	7.26 ± 0.02	NQ	9.46 ± 0.62	NQ	-
**11**	Benzaldehyde	1238	1234	NQ	33.48 ± 2.00	16.61 ± 0.83	34.23 ± 2.38	Citrus
**Esters**								
**12**	Isoamyl acetate	875	875	2.73 ± 0.30	6.12 ± 0.67	2.99 ± 0.33	8.25 ± 0.02	Banana, fruity
**13**	Ethyl hexanoate	1011	1000	3.85 ± 0.42	3.39 ± 0.19	3.39 ± 0.37	6.49 ± 0.03	Apple, Pineapple
**14**	Ethyl benzonoate	1187	1166	0.92 ± 0.12	NQ	1.32 ± 0.02	2.09 ± 0.23	Fruity, grape
**15**	Ethyl octanoate	1197	1197	2.66 ± 0.29	19.10 ± 1.04	20.17 ± 1.10	22.70 ± 0.89	Apricot, fruity
**16**	Phenethyl acetate	1243	1251	20.35 ± 1.11	22.33 ± 0.19	22.99 ± 1.25	27.83 ± 1.52	Fruity, floral
**17**	Ethyl 9-decanoate	1390	1385	2.39 ± 0.20	1.43 ± 0.23	1.12 ± 0.18	0.90 ± 0.00	Fruity
**18**	Ethyl deceanoate	1396	1395	2.22 ± 0.24	2.51 ± 0.27	2.75 ± 0.30	3.12 ± 0.05	Fruity, apple
**19**	Ethyl hexadecanoate	1994	1992	0.40 ± 0.06	1.85 ± 0.12	2.02 ± 0.22	2.68 ± 0.29	Fruity
**20**	Ethyl dodecanoate	1595	1591	NQ	1.19 ± 0.19	1.65 ± 0.27	0.99 ± 0.16	Sweet, waxy
**21**	Ethyl benzeneacetanoate	1243	1243	NQ	NQ	1.12 ± 0.18	0.55 ± 0.09	Floral, honey
**Terpenes**								
**22**	Linalool	1140	1100	18.06 ± 1.18	NQ	25.70 ± 1.40	NQ	Floral, citrus
**23**	Citronellol	1225	1228	23.58 ± 1.29	24.29 ± 0.54	24.57 ± 1.34	24.75 ± 1.25	Floral, roses
**24**	β-Damascenone	1392	1373	4.75 ± 0.35	NQ	NQ	NQ	Fruity
**Others**								
**25**	2-methoxy-4-vinylphenol	1314	1305	25.56 ± 1.39	26.55 ± 1.45	13.33 ± 0.87	9.42 ± 0.62	Woody
**26**	Cadalene	1671	1341	3.08 ± 0.34	1.67 ± 0.07	1.80 ± 0.30	NQ	-
**27**	γ-n-amylbutyrolactone	1371	1355	1.76 ± 0.29	1.98 ± 0.22	0.73 ± 0.12	0.26 ± 0.04	Coconut

LRIa: Literature Linear Retention Index (NIST); LRIb: Experimental Linear Retention Index; NQ (not quantified).

**Table 5:** Average scores given by panelists for sensory attributes of beers.

		Beers		
Attributes	Control (CS)	Beers with 20 g/L of Tamarind Pulp Added During Maturation (BTDM20)	Beers with 40 g/L of Tamarind Pulp Added During Maturation (BTDM40)	Beers with 60 g/L of Tamarind Pulp Added During Maturation (BTDM60)
Color	7.19 ± 1.46 ^a^	7.26 ± 1.56 ^a^	7.15 ± 1.45 ^a^	7.08 ± 1.37 ^a^
Aroma	6.85 ± 1.39 ^a^	7.05 ± 1.49 ^a^	6.69 ± 1.28 ^a^	6.77 ± 1.61 ^a^
Turbidity	6.90 ± 1.43 ^a^	6.84 ± 1.44 ^a^	6.77 ± 1.31 ^a^	6.44 ± 1.62 ^a^
Flavor	6.21 ± 2.04 ^a^	6.15 ± 1.93 ^a^	5.69 ± 2.10 ^a^	5.73 ± 2.30 ^a^
Appearance	7.16 ± 1.52 ^a^	7.26 ± 1.35 ^a^	6.95 ± 1.32 ^a^	6.77 ± 1.58 ^a^
Evaluation	6.89 ± 1.38 ^a^	6.84 ± 1.59 ^a^	6.47 ± 1.77 ^a^	6.13 ± 1.92 ^a^
Purchase intention	3.53 ± 1.22 ^a^	3.47 ± 1.28 ^a^	3.23 ± 1.22 ^a^	3.06 ± 1.27 ^a^

Results presented as average values. Equal letters on the same line indicate that the means do not differ statistically at *p* < 0.05 using the Tukey test.

## Data Availability

The original contributions presented in the study are included in the article, further inquiries can be directed to the corresponding author.

## Abbreviations

The following abbreviations are used in this manuscript:

CS	Beer without tamarind pulp
BTDM20	Beers with 20 g/L of tamarind pulp added during maturation
BTDM40	Beers with 40 g/L of tamarind pulp added during maturation
BTDM60	Beers with 60 g/L of tamarind pulp added during maturation
SM	Start of mashing
M70 °C	Temperature during mashing to 70 °C
M78 °C	Temperature during mashing to 78° C
BB	Beginning of boiling
EB	End of boiling
SF	Start of fermentation
M	On the seventh day of maturation
P	Packing
RB	Ready beer
Suc	Sucralose
Glu	Glucose
Fru	Fructose

## Appendix A

### Appendix A.1

**Table A1:** Physicochemical characterization of tamarind pulp.

Parameters	Values
pH	2.97 ± 0.02
TTA (g of tartaric acid/100 g of pulp)	5.32 ± 0.00
TSS (° Brix)	25.35 ± 0.14

Total titratable acidity (TTA); total soluble solid content (TSS).

### Appendix A.2

**Table A2:** Sugar concentrations in various beers analyzed by HPLC systems.

							Sugar (mg/mL of Beer)					
							Samples					
	Control (CS)			Beers with 20 g/L of Tamarind Pulp Added During Maturation (BTDM20)			Beers with 40 g/L of Tamarind Pulp Added During Maturation (BTDM40)			Beers with 60 g/L of Tamarind Pulp Added During Maturation (BTDM60)		
Stages	Suc	Glu	Fru	Suc	Glu	Fru	Suc	Glu	Fru	Suc	Glu	Fru
**SM**	69.56 ± 0.13	20.72 ± 0.58	NQ	NQ	NQ	NQ	NQ	NQ	NQ	NQ	NQ	NQ
**M70 °C**	77.92 ± 0.61	NQ	NQ	NQ	NQ	NQ	NQ	NQ	NQ	NQ	NQ	NQ
**M78 °C**	82.88 ± 0.08	NQ	NQ	NQ	NQ	NQ	NQ	NQ	NQ	NQ	NQ	NQ
**BB**	51.08 ± 1.40	NQ	NQ	NQ	NQ	NQ	NQ	NQ	NQ	NQ	NQ	NQ
**EB**	61.39 ± 0.10	24.04 ± 0.80	NQ	NQ	NQ	NQ	NQ	NQ	NQ	NQ	NQ	NQ
**SF**	22.12 ± 0.79	NQ	2.68 ± 0.02	NQ	NQ	NQ	NQ	NQ	NQ	NQ	NQ	NQ
**M**	3.52 ± 0.03 a	4.02 ± 0.05 d	NQ	3.44 ± 0.10 d	4.32 ± 0.09 d	NQ	6.10 ± 0.10 a	5.12 ± 0.44 c	NQ	6.20 ± 0.00 a	6.16 ± 0.02 b	NQ
**P**	NQ	5.84 ± 0.03 b	3.8 ± 0.05 d	NQ	5.64 ± 0.05 b	3.56 ± 0.02 d	NQ	6.32 ± 0.01 a	4.28 ± 0.02 c	NQ	6.44 ± 0.02 a	4.68 ± 0.01 c
**RB**	NQ	NQ	NQ	NQ	NQ	NQ	NQ	NQ	NQ	NQ	NQ	NQ

Results presented as averages. Equal letters on the same line indicate that the means do not differ statistically at *p* < 0.05 using the Tukey test; not quantified (NQ).
